# Visual-Aided Obstacle Climbing by Modular Snake Robot †

**DOI:** 10.3390/s24134424

**Published:** 2024-07-08

**Authors:** Carla Cavalcante Koike, Dianne Magalhães Viana, Jones Yudi, Filipe Aziz Batista, Arthur Costa, Vinícius Carvalho, Thiago Rocha

**Affiliations:** 1 Department of Computer Science, University of Brasília, Brasília 70910-900, Brazil; 2 Department of Mechanical Engineering, University of Brasília, Brasília 70910-900, Brazil; diannemv@unb.br (D.M.V.); jonesyudi@unb.br (J.Y.); viniciuscgomes.98@gmail.com (V.C.); dedeusthiagounb@gmail.com (T.R.); 3 Mechatronics Graduate Programme, University of Brasília, Brasília 70910-900, Brazil; filipe.aziz@aluno.unb.br (F.A.B.); arthur.reichert@aluno.unb.br (A.C.)

**Keywords:** snake robots, climbing robots, image-guided locomotion

## Abstract

Snake robots, also known as apodal robots, are among the most common and versatile modular robots. Primarily due to their ability to move in different patterns, they can evolve in scenarios with several constraints, some of them hardly accessible to other robot configurations. This paper deals with a specific environment constraint where the robot needs to climb a prismatic obstacle, similar to a step. The objective is to carry out simulations of this function, before implementing it in the physical model. To this end, we propose two different algorithms, parameterized by the obstacle dimensions determined by image processing, and both are evaluated in simulated experiments. The results show that both algorithms are viable for testing in real robots, although more complex scenarios still need to be further studied.

## 1. Introduction

The locomotion of robots in unstructured environments is one of the main challenges for the insertion of mobile robotics in a wide variety of real-world applications. Unknown environments can impose very different obstacles to those encountered in well-structured environments such as laboratories, factories, hospitals, and commercial buildings. In activities such as environmental monitoring, precision agriculture, mining and cave exploration, and search∖rescue operations, the environment can be dynamic, uncontrollable, and often not previously mapped [1,2].

In order to overcome the challenges of locomotion in unknown environments, robots must be versatile, flexible, and able to overcome different obstacles. The mechanical structure of a mobile robot is directly related to its goal tasks and the environment where it is supposed to evolve, and no single mechanical structure can move efficiently in all kinds of terrain. Modular robots have been studied as viable alternatives for this type of environment because they can reconfigure their form of locomotion according to the constraints of the environment [3,4].

Nature solutions for this problem have inspired mechanical designs that mimic snakes due to their ability to adapt their locomotion patterns according to the environment. Snake robots are multi-redundant, built mainly by a collection of similar, but not always identical, modules. The adaptability of snake robots makes them good candidates for several applications [5].

Among the advantages of snake robots can be cited their adaptability, their variety of locomotion gaits, and their ability to move in narrow spaces. However, there are also some downsides, like the mathematical complexity due to the high number of degrees of freedom and the limitations in space, power, and communication, which restrict the amount and type of sensors [6,7].

In medical robotics, the possibility of having different tools as modules offers the flexibility for the use of snake robots for a variety of medical procedures like endoscopy surgery [8], organ mapping [9], and many others [10]. In extreme environments (after disasters, toxic environments, or search and rescue), the snake robot’s ability to navigate on complex terrain can present important benefits. In these situations, the ability to climb obstacles to pass over them is a considerable challenge to wheeled robots, and snake robots can be designed and controlled to achieve it [11]. The advantages of snake robots in tasks like search and rescue, and land exploration, where the robot is expected to move among the rubble and in confined spaces, derive from their aptness to adapt their locomotion patterns depending on the environment characteristics [6].

Adaptability is also an asset when the robot is expected to climb obstacles, as the dimensions, distance, and shape of these obstacles may demand different approaches to surmount them. A diversity of strategies can be used by a snake robot to surpass obstacles [12]. When climbing over an obstacle, a snake robot can raise some of its modules to become higher than the obstacle, a technique known as free climbing. However, the robot can also perform aid (or aided) climbing when it uses supports like poles or slopes to move up (or around) the obstacle [6,11,13].

In order to navigate, the robot needs to have some level of knowledge about its surroundings, as well as its orientation/localization. In unknown environments, using intrinsic and extrinsic sensors is essential so that the robot can identify possible trajectories and overcome obstacles [14,15]. Among the various types of sensors, visual sensors (cameras) make it possible to extract a multitude of geometric information about the environment. They are a versatile alternative to other sensors such as ultrasound, lidars, infrared, and tactile [16].

This research work aims to develop and validate a modular snake robot able to climb obstacles. This paper addresses the simulation analysis that precedes the implementation in the physical model.

Equipped with a monocular camera, the robot detects obstacles and extracts their parameters using the Hough transform, a classical computer vision technique [17], as a pre-processing step for identifying lines in a binary image. The obstacle parameters are inputs for two parameterized object climbing algorithms depending on the robot’s initial position to the obstacle. The first algorithm is used for frontal climbing, and the second is for lateral obstacle climbing [18]. The robot can position itself in the best way to climb, depending on which algorithm is most easily applicable to each situation.

The mechatronic design of the modules is explained in detail, as well as their construction and prototyping aspects, followed by the selection, implementation, and testing of the computer vision techniques used. Finally, the climbing algorithms are explained in detail. Tests in a simulated environment were used to stress each component of the robotic system: modular mechanical aspect, computer vision, and locomotion algorithms.

### 1.1. Related Work

#### 1.1.1. Modular Snake Robots

In 1972, the first modular snake robot was developed by Hirose [19], called the Active Cord Mechanism (ACM), consisting of multiple modules (compartmentalized sections). It was designed with a high degree of redundancy, forming a highly reliable system. Hirose and Mori engineered the modules to be independent, allowing them to be replaced or removed without compromising the whole structure. Moreover, sections of the snake robot could be separated to perform different tasks simultaneously [19].

The movement of Hirose and Mori’s ACM followed a “serpentine curve” motion pattern on the ground plane, enabling forward and backward movement but lacking vertical motion. In certain environments, moving laterally was found ineffective as the robot struggled to navigate around some types of obstacles [12].

Hirose’s research on biological snakes revealed that movement extended beyond being merely two-dimensional. As different body sections were sequentially lifted, the points of contact varied, enhancing motion efficiency. He found it advantageous to minimize counterproductive drag friction. His conclusion emphasized the necessity of vertical mobility for precise mimicry [19].

Hirose focused on control theory, independently developing actuators with individual control. Sensors on each actuating joint provided feedback regarding the joint’s angular position. These actuators could adjust their response based on the overall motion plan by utilizing angle feedback, covering characteristics such as amplitude, wavelength, and velocity of the snake’s motion [19].

Several years after Hirose, Chirikjian studied snake robot control using a geometric approach to motion planning, focusing on planar kinematics formulation [20]. Ostrowski and Burdick continued motion planning studies using geometric mechanics [21].

In 2004, Hirose and Mori introduced a new proposal for the modular snake robot, incorporating various movements beyond the horizontal plane. The new robot, named ACM-R3, featured serpentine motion, sinusoidal lifting, pedal wave, lateral coiling, and, finally, an aquatic spiral motion [22].

Through extensive studies, the necessary characteristics for a snake robot were defined [23]. The first characteristic considered was a small cross-sectional area, a bodily feature common in biological snakes, that allows the robot to perform tasks in narrow environments. The second characteristic is hyper-redundancy, where robots have many degrees of freedom, enabling them to perform tasks of varying complexities. Underactuation is the third necessary characteristic, meaning they have fewer control inputs than degrees of freedom. The fourth characteristic is robust locomotion, enabling the robot to move in various environments, regardless of the local structure. Lastly, the fifth characteristic is having manipulator vehicle properties, allowing the robot to transport objects and be used as a manipulator.

**Figure 1:**
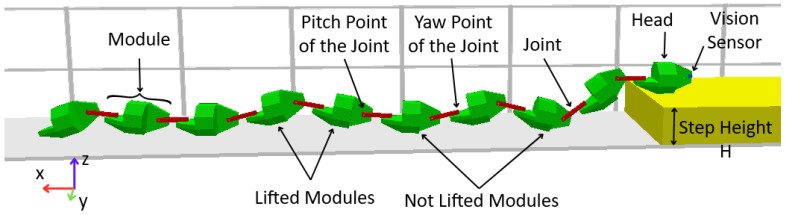
Modular snake robot employed in this work.

This project was based on the defined characteristics necessary for a snake robot, addressing other aspects to be discussed in future sections. Figure 1 illustrates the snake robot employed as a base for the experimental part of this work. It features 10 identical modules; this amount can be increased, as needed, for other tasks. The figure shows that between each pair of modules there is a joint. The piece that represents the joint has two ends, on one of which it is possible to perform the pitch movement and on the other the yaw movement. A visual sensor is attached to the first module, referred to as the head module.

#### 1.1.2. Obstacle Climbing

Regarding indoor environments, stairs pose one of the most challenging scenarios for service robots. There are multiple materials, geometries, and techniques used to build stairs, which may vary even from one step to the other. Therefore any robot that intends to climb one or more steps reliably is required to have a carefully designed locomotion algorithm, which may be aided by sensors. According to the employed locomotion method, there are four main types of stair-climbing robots: tracked, legged, wheel-legged, and wheel-linkage robots. They are, by far, the most commonly used, because of their fast climbing and known kinematic and dynamic control possibilities [24].

Tracked climbing robots are a good choice to overcome the shape or size limitations of the stairs. Their large contact surface makes them less susceptible to tumbling, especially with steps whose dimensions or shapes are unknown. PackBot [25] is an example of a tracked robot that has been used for chemical and radiation hazard detection and even human battlefield casualty extraction, including step climbing. Like many other tracked robots, it does not perform well in riser-less steps or those with huge noses. On the other hand, legged robots perform well in most step scenarios, at the expense of a highly complex path planning algorithm to mimic biological moving patterns or other optimized climbing gaits, as performed, for example, in a RHex-style hexapod robot [26], which was able to climb a step 3.9 times higher than its own leg using a quasi-static climbing gait. As for wheel-linkage robots, their ability to climb is directly tied to the existence of the stair nose and riser. They are suited for uneven and rubble terrain but also rely on costly computational power to generate optimal gaits [27]. Lastly, wheel-legged robots tend to offer a balanced velocity output, both on flat terrain and over obstacles. These robots are also suited for space exploration missions if their maneuverability is tied to a lightweight design such as GerWalk [28].

However, snake robots are able to traverse almost any obstacle because of their extensive environmental body compliance. In comparison to other means of climbing, there have been few case studies regarding snake robots. One of the first works about snake robots and obstacle climbing [11] discussed the relation between the free climbing requirement of torque and the design of the robot.

Nilsson [11] was also the first to classify climbing approaches as free or aided. ACM-R8 is an example of an active-wheel snake-like mechanism that is designed to climb uneven terrain, specially stairs. Traditionally, wheeled robots can only traverse steps with riser heights smaller than the diameter of its wheels. This, however, does not hamper ACM-R8’s ability to climb, because of its serial modular connection together with its swing grousers, allowing it to go beyond normal wheeled robot limitations [29]. There are also robots that couple the locomotion benefits of snake-like mechanisms and the gripping benefits of manipulator arms. The T² Snake-4 pitch–yaw connection robot encompasses both characteristics and together with a wireless multi-sensor control is able to climb steps upwards using a simple gait generator known as a single backward wave, also aided by groused passive wheels. Its manipulation capabilities enable its use in many situations such as agriculture and industrial inspection, both in structured and unstructured environments.

In many rotational mechanisms, there is a known difficulty in going over a step without the collision of the mechanism and the step’s edge. The use of prismatic joints is regarded as one of the possible solutions to this issue, especially if torque minimization is a key feature to be considered [30].

The use of a laser range finder sensor attached to the head of the snake robot has been shown to be able to identify key step features that could be used to enhance path planning algorithms to climb steps while also keeping track of sensible variables such as total system torque and energy consumption [31]. Aided by passive wheels, this robot achieved step climbing with bounded height obstacles. The use of mechanical solutions can greatly improve system performance with low production and use costs. By using passive ratchet wheels, a snake robot can greatly improve its climbing functionalities regarding its forward and backward axis (from tail to head) with friction anisotropy, which is also useful to perform gaits with more stability [32]. This set of references showcases the huge adaptability of snake robots to variable scenarios that may pose difficulties to more common climbing robots. The use of wheelless snake robots, or apodal snake robots, might be useful to broaden the already huge locomotion possibilities of wheeled robots, but with fewer constraints regarding wheel diameter, for example.

In order to investigate locomotion possibilities for an apodal snake robot, this work proposes the use of a visually aided obstacle climbing procedure to enable two different climbing gaits aided by a sinusoidal generator joint control scheme that is easily implementable in low-cost micro-controllers.

#### 1.1.3. Visual-Aided Navigation

Navigation and planning is essential in the execution of complex tasks in mobile robotics, especially in complex and constantly changing environments, such as those often found in disaster relief operations in which the use of snake robots is potentially advantageous [23]. In constantly changing scenarios, prior information is not reliable and has to be combined with measured data. Therefore, the problem becomes that of simultaneous localization and mapping (SLAM). Many frameworks allow the incorporation of measurements into prior data, especially Bayesian filtering and estimation techniques [33,34]. Proper measurements have to be obtained from range sensors, cameras, and other available hardware.

The problem of measurement consists in obtaining geometrical information concerning the environment from varied sensor data. One commonly applied technique is that of structure from motion (SFM), which allows the acquisition of a point cloud of the surrounding environment through a series of images taken at different positions and orientations (robot poses). There is a huge variety of SLAM techniques such as ORB-SLAM, direct sparse odometry, LOAM, and ART-SLAM. The choice for each one of them depends on the environment’s geometrical and photometric distortions, as well as the availability for sensor fusion [35]. In civil construction, sensor fusion has been used to detect and reconstruct a 3D map of external cracks assessing structural integrity in possibly cluttered and unstable environments [36].

Knowledge of the required information can be used by path planning to achieve poses in which more information can be inferred by the images captured, such as proposed in [37]. In simpler tasks, mathematical transformations such as the Hough transform can be employed to the input data to treat the problem in a more concise domain of parameters. Images often contain much unnecessary information such as color- and texture-related data. Naturally, the same can be said about range information and other kinds of sensory input, which might not be presented in the most adequate format. Pre-processing techniques allow the use of simpler algorithms, such as reported in [38], in which the Hough transform was applied to a set of points obtained through range data.

The problem of path planning regarding the previous use of SLAM-based techniques has also been addressed. In a controlled environment, perception-driven obstacle-aided locomotion (POAL) was achieved with the aid of both tactile and visual sensors. This sensor fusion reliably improved pushpoint detection [13]. In an unstructured orchard, the IKB-RRT path planning algorithm was proposed, which reduced both computation time and path length while also regarding path smoothness, obstacle avoidance, and the constraints of the tested non-holonomic robot [39].

In the current work, the very specific problem of step climbing is addressed through a custom-tailored algorithm that employs line parameters obtained through the Hough transform to identify the height of the step and its distance to the robot that will climb it. The visibility of the step and its geometry are assumed as prior information in this work.

## 2. Material and Methods

The methodological approach is divided into four basic steps. First, the design of the robotic system was carried out, which involved the development and construction of the module, detailing the mechanical and electronic components, and defining the boundary conditions and the target environment for the obstacle climbing tests.

Next, visual sensing was implemented, considering the use of a monocular camera to capture images of the environment. The Hough transform was applied for image processing and extraction of geometric parameters of obstacles.

Two distinct locomotion algorithms were developed for obstacle climbing: one based on pitch–pitch movements and the other on pitch–yaw combinations. These algorithms were described in detail, including the initialization of the robot’s position, the capture of the images, and the execution of the climbing movements.

Finally, simulations and validations were conducted using the CoppeliaSim simulator, which allowed testing and validation of the developed algorithms.

These steps are detailed in the following subsections, providing an overview of the development and validation process of the proposed system.

### 2.1. Robotic System Design

The following sections describe the robot’s physical design, as well as the software and proposed algorithms.

#### 2.1.1. Robot Design

**Figure 2:**
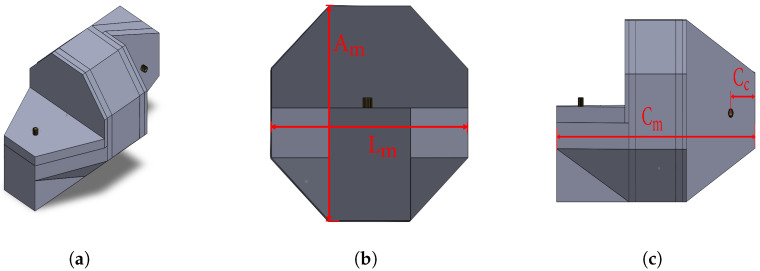
Module’s dimensions and overall geometry. (**a**) Module isometric view; (**b**) width and height of the module; (**c**) module’s length and distance to motor axis.

It is essential to have a simple, robust, and physically compliant modular mechanism design to enhance the robot’s locomotion efficiency in terms of displacement and velocity, and provide an adequate locomotion strategy. Figure 2 displays three images: Figure 2a displays the isometric view for the chosen module, Figure 2b shows its height, $A_{m}$, and width, $L_{m}$, and Figure 2c depicts each module’s full length, $C_{m}$, and the overlapping distance, $C_{c}$, when two modules are directly joined. Each of the described dimensions is set to $A_{m}=L_{m}=80$ mm, $C_{m}=155.8$ mm, and $C_{c}=21.25$ mm. The chosen module, previously presented in [18,40], is known as Erekobot *Neke*.

The outer surface of each module of the robot comprises three distinct features. Firstly, a regular octagonal prism serves to deter inadvertent rolling motions. The remaining two surfaces feature unconventional geometries, similar to a chamfered trapezoidal prism, each positioned adjacent to one of the octagonal prism’s bases.

**Figure 3:**
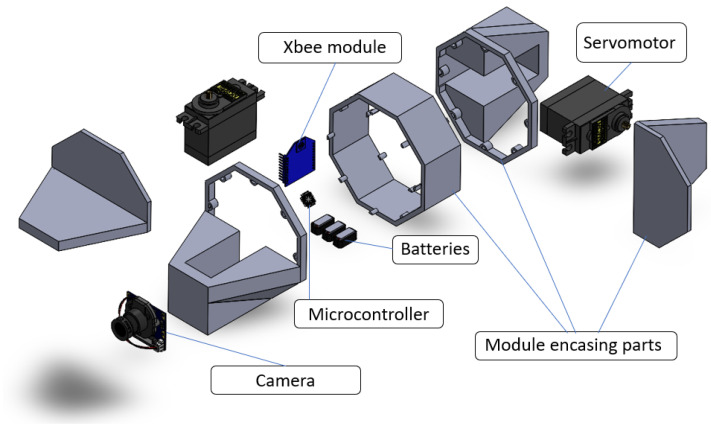
Module’s components and assembly.

Figure 3 shows part of the internal instrumentation embedded in Erekobot *Neke*. Enclosed within this central prism are essential components such as the Arduino microcontroller, a battery, a power board, and an Xbee module. Every module is outfitted with two servomotors tasked with moving the robot, one for each non-traditional geometry surface. Additionally, within the same module, one surface with a non-traditional geometry is consistently oriented 90 degrees apart from its counterpart, facilitating yaw and pitch movements. The complete module assembly weighs 3 Newtons, with each motor configured to exert a torque of 3 Newton meters.

Due to the chosen locomotion algorithm, each module’s dimensions play an essential role in the robot’s displacement. Due to the general sinusoidal generator approach of locomotion, dimensions $C_{c}$, $C_{m}$, $L_{m}$, and $A_{m}$ are direct entries to the computing of the overall displacement function.

The entire robot assembly is performed by interconnecting modules and linking rods in a serial orientation. These rods measure $C_{h}$ in length and couple one module’s pitch joint with the next module’s yaw joint, enabling these servos to take turns moving the robot and creating a functional two-degrees-of-freedom module.

#### 2.1.2. Targeted Environment and Boundary Conditions

In unknown and unstructured environments, the robot will encounter different types of obstacles as it navigates. This variability poses many challenges for mechatronic design, computer vision techniques, and locomotion algorithms.

This work considers the simplified problem of climbing an abrupt elevation, a step between two parallel planes. In order to test the robot’s locomotion algorithms, a parameterizable height and width step were selected.

Our algorithms expect the step to be horizontally flat and rectangular with no rubble on top of the steps. It is assumed that the dimensions of the step are $S_{h}\times S_{w}\times S_{L}$, where $S_{h}$ represents the height, $S_{w}$ represents the width, and $S_{L}$ represents the length. The steps are not required to have fixed dimensions, and this variability choice provides a more versatile working environment for the robot, enabling it to succeed in a broader obstacle dimension range.

#### 2.1.3. Visual Sensing

The snake robot’s planned trajectory to overcome a given obstacle (for example, climbing a step, as illustrated in this work) depends on geometrical parameters such as its length, height, distance to the obstacle, and the obstacle’s height, among others. While some of the needed values are intrinsic to the robot’s construction and configuration, others are extrinsic, and must be measured on a case-by-case basis.

Sensing and perceiving the external environment can be achieved through multiple strategies, such as force sensing, range sensors, and camera-based techniques [7]. Out of those, vision is ubiquitous in the natural realm and is consistently employed by varied creatures. Similarly, a snake robot can use cameras and computer vision algorithms to aid in path planning and navigation, enabling it to acquire the necessary information.

In the present work, we assume the snake robot is traversing a flat terrain towards a step that consists of a rectangular prism. We assume that at least one edge of each visible face is contained entirely within a plane perpendicular to the camera. Furthermore, we consider the robot’s displacement to possess a non-zero component along the direction perpendicular to the camera plane (the direction pointed by the camera at the initial time) and that the camera returns to that orientation at a second moment. Two images are captured and compared, one at an initial time and another after a displacement.

The constraint that the two images are taken with the camera at the same orientation is flexible, given that the robot can reorient the module containing the camera to meet the requirements. As for the displacement, since the robot needs to be near the step to climb it, it is only expected that the displacement will have a component perpendicular to the image plane if the obstacle is visible.

**Figure 4:**
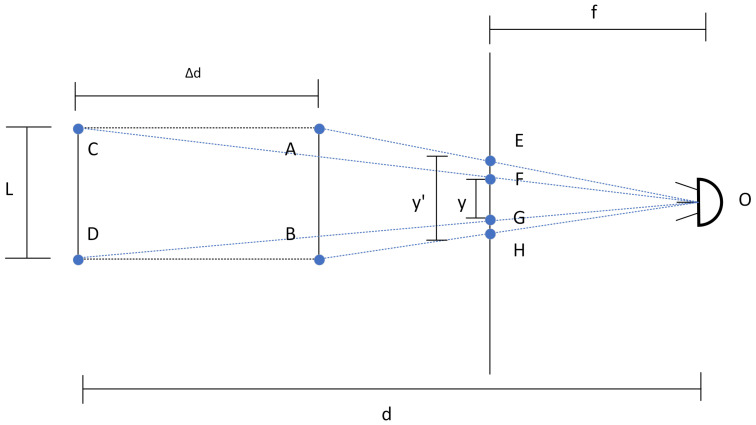
Schematic view of the projected lengths according to the distance to the observer and size of the segment.

As expected, the concepts above can be equally illustrated by an approaching obstacle and a fixed observer. Figure 4) illustrates the case of a line segment entirely contained within a plane parallel to the image plane and approaching the camera with a constant length. The focal distance of the camera is denoted by *f*, *d* is the initial distance of the object to the camera, Δd is the modulus of the displacement, *L* is the length of the segment, and *y* and y′ are the projected lengths of the segment.

Analysing the image, through the congruency between triangles ΔOFG and ΔOCD, and triangles ΔOEH and ΔOAB, one can arrive at the following relations:

(1) $$\begin{array}{c} \frac{y}{f}=\frac{L}{d},\;and \end{array}$$

(2) $$\begin{array}{c} \frac{y^{\prime }}{f}=\frac{L}{d-\Delta d}, \end{array}$$

which can be written as

(3) $$\begin{array}{c} d=\Delta d\left(\frac{y^{\prime }}{y\prime -y}\right),\;and \end{array}$$

(4) $$\begin{array}{c} L=\frac{\Delta d}{f\left(\frac{1}{y}-\frac{1}{y\prime }\right)}, \end{array}$$

where the unknown terms *d*, and *L* are expressed in terms of known quantities.

The snake robot can determine the distance to an obstacle and its height through the previously derived expressions. That being defined, identifying the obstacle in the image requires applying the technique described. In order to locate a line segment through which the appropriate measurements are taken (height and distance), the proposed algorithm relies on identifying and matching in both images the convex quadrilateral corresponding to the face of the step nearest to the robot.

To locate convex quadrilaterals in a given image, an edge detector (a canny edge detector in this work) is applied, and the resulting output is fed to a Hough transform algorithm to locate lines represented in the image [17]. The information on the lines in the image domain is used to find quadrilaterals. First, all pairs of lines that intersect in the domain (all lines that are not parallel intersect, not necessarily in the image domain though, and the intersection might not be drawn in the image) are located. This information is used to build an unweighted, undirected graph in which the nodes are the lines, and the vertices are connected by edges whenever two lines intersect in the image domain. It follows from the definitions that the neighborhood of a node, defined as $N\left(u\right):=\left\{v\in V:\{u,v\}\in E\right\}$, where *u* and *v* are nodes in the set of nodes *V* and *E* is the set of edges, represents the set of all lines that intersect a given line in the image domain.

A quadrilateral necessarily possesses exactly four vertices connected by line segments, in which no more than two vertices belong to the same line segment forming the quadrilateral. In the earlier graph representation, vertices can be identified with the edges representing intersections between lines. All quadrilaterals form closed trails of length 4 in the graph representation of the lines and their intersections in the image (a closed trail of length 4 in the graph might not be associated with a quadrilateral though, since two different edges might be associated to the same vertex, such as the case of three lines that all intersect in the same point). Therefore, the problem of identifying quadrilaterals in the image is equivalent to finding closed trails of length 4 in the constructed graph, where each edge is associated with distinct points in the image.

**Figure 5:**
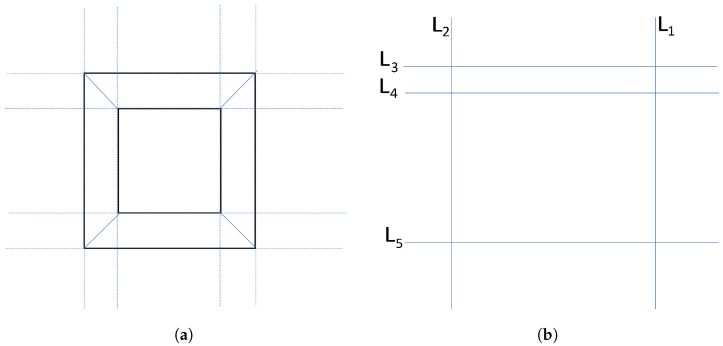
Source image and processed image after edge detection and Hough transform. (**a**) Source image; (**b**) detected lines in pre-processed image.

To illustrate these ideas, let us consider an example. Figure 5a is an example of an image containing some lines that represent existing lines in the image captured by the camera. It is considered that there may be more than one rectangle in the image besides the face of the step, so it is necessary to distinguish the step from any other rectangles in the image. Figure 5a exemplifies an image in which there is a step (outer rectangle) and another extra rectangle. On the other hand, Figure 5b represents the lines detected after pre-processing carried out in Figure 5a where not all lines were detected.

**Figure 6:**
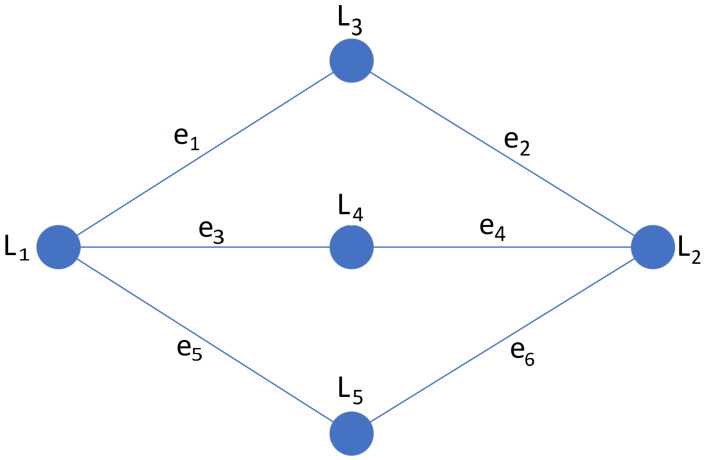
Resulting undirected unweighted graph.

Applying the procedure described to build a graph where the nodes are the lines detected, and every intersection between two lines in the image domain is connected by an edge, we obtain the graph illustrated in Figure 6.

All quadrilaterals in Figure 5b are equivalent to the trails in the graph in Figure 6, defined by the sequences of vertices $\left(l_{1},l_{3},l_{2},l_{4},l_{1}\right)$, $\left(l_{1},l_{3},L_{2},l_{5},l_{1}\right)$, and $\left(l_{1},l_{4},l_{2},l_{5},l_{1}\right)$, which imply the sequences of edges $\left(e_{1},e_{2},e_{4},e_{3}\right)$, $\left(e_{1},e_{2},e_{6},e_{5}\right)$, and $\left(e_{3},e_{4},e_{6},e_{5}\right)$, respectively. Since each edge is associated with a vertex, if no two edges are associated with the same vertex (which means the mapping between edges in the graph and points in the image is injective in the set of edges of the path), the closed path of length 4 in the graph is equivalent to a quadrilateral in the image. The quadrilateral can be traced by connecting the points mapped to the edges in sequence. In this sense, each quadrilateral can be described by a sequence of four distinct points in the image, which are connected in sequence to form it. Note that two distinct sequences containing the same points can be equivalent to the same quadrilateral (the inverse is not always true; given a permutation, the points may be the same, but the new sequence formed might not describe the same quadrilateral).

The Algorithm 1 is proposed for identifying the quadrilaterals (a set of ordered sequences of points) in the image given the graph G=(V,E), where *V* is the set of nodes and *E* the set of edges built as described:
**Algorithm 1** Quadrilaterals identification algorithmQuadSet←{}             ▹ Initialize the set of quads as an empty set.**for **$n_{i}\in V$**do**    **for** $\left(n_{j},n_{k}\right):n_{j}\neq n_{j},n_{j},n_{k}\in N\left(n_{i}\right)$**do**▹ Pairs of nodes in the neighbourhood of $n_{i}$        $\mathrm{exclusion}\leftarrow \{n_{i},n_{j},n_{k}\}$       ▹ The set of nodes already accounted for.        $\mathrm{SetOfOpposites}\leftarrow \{\left(N\left(n_{j}\right)\setminus \mathrm{exclusion}\right)\cap \left(N\left(n_{k}\right)\setminus \mathrm{exclusion}\right)\}$        **for** $o_{i}\in \;$SetOfOpposites **do**           $\mathrm{QuadSet}\leftarrow \mathrm{QuadSet}\cup \{\left(n_{i},n_{j},o_{i},n_{k}\right)\}$        **end for**    **end for**    Remove node from the graph**end for****return **QuadSet

Once all quadrilaterals in both images have been identified, all that is left is to select two matching quadrilaterals (i.e., that describe the same elements in world space), one in each image, that represent the face of the step nearest to the snake robot in the environment. Noting that the ordering of quadrilaterals according to the area (that is, the ranking from smallest to largest) is preserved if the elements visible in both images are the same and the orientation is kept unchanged, in this work we select, in each image, the quadrilateral with the largest area.

### 2.2. Climbing Algorithms

Each of the climbing scenarios can be divided into 2 different, but complementary phases: crawling and climbing. For each algorithm, there is a chosen initial position and orientation from which the movement begins. The initial position is defined by the procedure initialize_algorithm(). In the case of the first algorithm, the robot must begin its locomotion with its body longitudinally parallel to the step’s width, but for the second algorithm the initial setup requires that the robot is parallel to the step’s length. Both algorithms require alignment of the robot with the step and, for this, it is possible to rotate the complete robot in the plane around the z-axis using the simulation of serpentine movement [41,42,43].

From the initial condition, the robot crawls according to auxiliary Algorithm 2. This algorithm is used to guide the robot towards the chosen head position in order for the climbing algorithm to be executed, which is geometrically based. By choosing K∈Q as the number of undulation cycles, the robot proceeds to crawl toward the step. After *K* cycles, it aligns the camera to face the step riser orthogonally and then acquires an image that is then processed as described in Section 2.1.3. With the new data input from the image processing, it is possible to calculate how many cycles are required to achieve the chosen initial head position so the climbing algorithm can begin.
**Algorithm 2** Image capture auxiliary algorithm initial_orientation←initialize_algorithm()**Require**: robot is set longitudinally parallel to initial_orientation; **while** not enough distance **do**     Crawl forward K cycles;              ▹ K choice is initially arbitrary     **if** head is not parallel to step **then**         Align head to step;     **else**         Acquire step picture;         Realign head to former position;         Determine remaining distance and step parameters;         Update crawling algorithm;     **end if** **end while** Update climbing Algorithm 1; Run climbing Algorithm 1;

#### 2.2.1. First Algorithm

In the first scenario, the robot’s body is longitudinally set perpendicular to the stair, and using only pitch movement it is expected to climb up the obstacle, one module at a time, so that throughout the simulation many of its modules are placed on top of the stair. In this case, the pitch movement is what both rises the robot up to the step’s superior edge and also places each module back on its superior surface.

The first climbing algorithm, also based on pitch-only locomotion, works according to Algorithm 3. It relies on previous information collected by Algorithm 2 to climb, one step at a time, one of its *N* modules at a time.

Previous work described in [18] displayed a set of geometrical constraints summarized by Equations (5)–(7). More specifically, the height, $S_{h}$, of the step was constrained by the robot’s dimensions. Because the robot was positioned perpendicularly to the step as the climbing algorithm was set up, the step width, $S_{w}$, and the robot length were also related. Similarly, the robot width and the step length, $S_{L}$, were also related. Lastly, the distance that the robot was capable of moving up its modules via pitch joint and the step height were also related.

The integration between Algorithms 2 and 3 allows the previous set of restrictions to be more flexible in terms of step height, although it still requires that the robot’s full length does not exceed the step’s width, $S_{w}$. The restrictions can be mathematically described as:

(5) $$S_{h}\leq \frac{L_{m}}{2}+C_{m}-C_{c},$$

(6) $$S_{w}\geq N\cdot C_{m}+N\cdot \left(C_{h}-2\cdot C_{c}\right),\;and$$

(7) $$S_{L}\geq L_{m},$$

where $C_{m}$, $C_{h}$, and $C_{c}$ are as shown in Section 2.1.1.
**Algorithm 3** Pitch–Pitch only step climbing**Require**: robot is set longitudinally perpendicular to the stair;**Ensure:** the step’s height has been acquired; Calculate L modules to rise; **for** i=N; i≤L; i−−**do**             ▹ Pushing modules up     Raise i^th^ module;     Approach; **end for** **do**                       ▹ Pulling modules up     Approach;     Place n^th^ module on top of the step;     n←n−1; **while** n>1**Ensure:** the robot is correctly positioned;

#### 2.2.2. Second Algorithm

This second scenario contains both the same obstacle and robot, but their relative orientation is now different. The initial configuration is set in a way that the robot is parallel in head-to-tail length to the step length, $S_{w}$. After displacing its body to the initial climbing position according to Algorithm 2, the robot is able to execute the appropriate climbing algorithm. Using both yaw and pitch joints, it is expected that the robot places as many of its modules as possible on top of the obstacle. Due to its initial configuration, pitch joints will raise each module until step height is achieved, and then yaw joints will laterally move each module until they are fully placed on top of the step. This procedure can be summarized in Algorithm 4.

Because of the different orientations between the robot and the step in this scenario, restrictions between modules and step dimensions are different [18]. The height of the step, $S_{h}$, is tied to the maximal height the robot is capable of achieving. The step width, $S_{w}$, is tied to the robot’s upper base surface. The step length, $S_{L}$, is tied to the robot’s total length. Equations (8)–(10) show these constraints. However, the choice of coordinating both Algorithms 2 and 4 also allows for flexibility, specially concerning the step height.

(8) $$S_{h}\leq \frac{A_{m}}{2}+C_{h}+C_{m}-C_{c},$$

(9) $$S_{w}\geq \frac{L_{m}}{2}+C_{h}+C_{m}-2\cdot C_{c}+\frac{L_{m}}{2},\;and$$

(10) $$S_{L}\geq N\cdot C_{m}+N\cdot \left(C_{h}-2\cdot C_{c}\right),$$

where $C_{m}$, $C_{h}$, and $C_{c}$ are as shown in Section 2.1.1.
**Algorithm 4** Pitch–Yaw step climbing**Require**: robot is set longitudinally parallel to the stair;**Ensure**: the creation of a ground-level foundation for the robot; Raise nth module; Raise (n−1)th module; Place nth module on top of the step; **do**     Place (n−1)th module on top of the step;     n←n−1 **while** n>6             ▹ Number of stable foundation modules Undo ground level foundation; Create a new foundation on top of the step; **do**     Place (n−1)th module on top of the step; **while** n>1 Undo the foundation on top of the step;**Ensure**: the robot is correctly positioned;

## 3. Results

Some experiments were carried out to test the methods proposed. For the movement described in Section 2.2, the CoppeliaSim simulator was chosen to verify the validity of the exposed theory. The version 4.3 for Ubuntu was used. The CoppeliaSim software (https://www.coppeliarobotics.com/), formerly known as VREP, is a simulator that uses inverse and direct kinematics and several libraries that help keep the physics characteristics of the models as faithful as possible to reality [44].

To investigate the theory exposed on computer vision, initially, a code was created in Python language for carrying out the calculations proposed in Section 2.1.3, considering the existence of two images photographed in such a way that the camera was parallel to the step and considering that the photos were taken at different distances from the step. To validate the code created, a second code was also made in Python, which simulates the view of a camera that approaches a virtual step, considering all the primary camera’s parameters necessary for the proposed calculations. Such parameters are described in Algorithm 1.

To investigate the association between the two theories, we decided to use the CoppeliaSim software, which accepts programming in Python and Lua languages and presents the possibility of using sensors in the simulation, such as the vision sensor, capable of capturing images. Therefore, it is possible to simulate the desired movement for the robot, using a camera and image processing during the movement to calculate the parameters necessary to climb the step ahead, all in an automated way.

### 3.1. Image Processing

**Figure 7:**
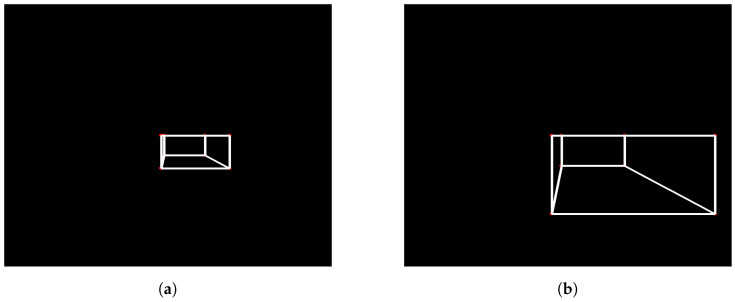
Photos obtained from a camera simulation code. (**a**) Camera first photo—simulation code; (**b**) camera second photo—simulation code.

As previously mentioned, a Python code was created to replicate photos taken by a camera advancing toward the step in a parallel manner. Figure 7a,b show two photos obtained with the mentioned code, where Figure 7a represents the first photo taken, with more distance from the step, and Figure 7b represents the second photo taken, closer to the step. It is possible to notice that the face of the step facing the camera is always parallel to the camera, an important point to be taken into consideration for the image processing step.

**Figure 8:**
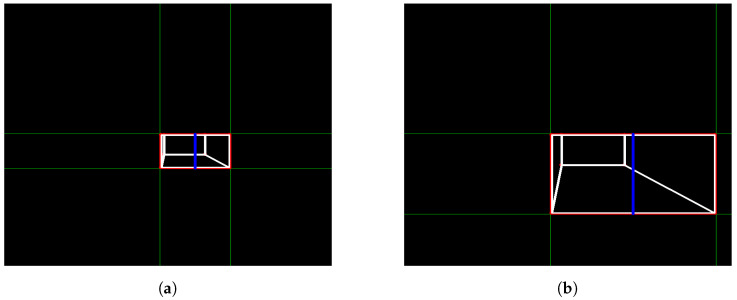
Analysis obtained Through image processing algorithm. (**a**) Camera first photo analysis; (**b**) camera second photo analysis.

When testing the image processing algorithm on the photos seen in Figure 7 and Figure 8a,b were obtained. These figures show the straight lines that intersect each other two by two at the vertices of the rectangle that defines the front face of the step. As explained in Algorithm 1, the straight lines found allow the identification of the four sides (edges) of the rectangle that form the front face of the step, allowing the calculation of the height, H, of the step, represented by the blue line. The result calculated by the algorithm was a step distance of 61.6907 measurement units and a height of 326.9605 measurement units, with the pre-established measurements in the photo simulation of the step being the distance to the step of 60 measurement units and a step height of 300 measurement units. Therefore, it can be seen that the calculation error reached was 0.4 percent, showing a small and acceptable error, especially taking into account that, for all images generated and tested, the error obtained would not hinder the movement of the robot if it were to occur in the real scenario.

An analysis was performed, running the simulation with different combinations of height and distance in order to evaluate the behavior of the measurements and the error with different values, all of which respected the constraint that the step was visible at all times. Table 1 shows the results obtained.

**Figure 9:**
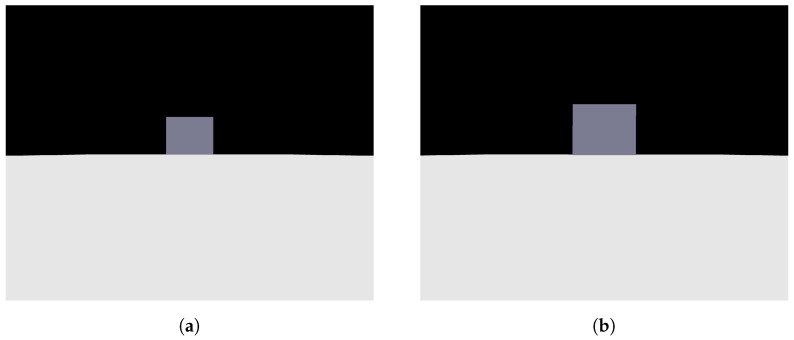
Photos obtained from vision sensor of CoppeliaSim. (**a**) Vision sensor first photo; (**b**) vision sensor second photo.

**Figure 10:**
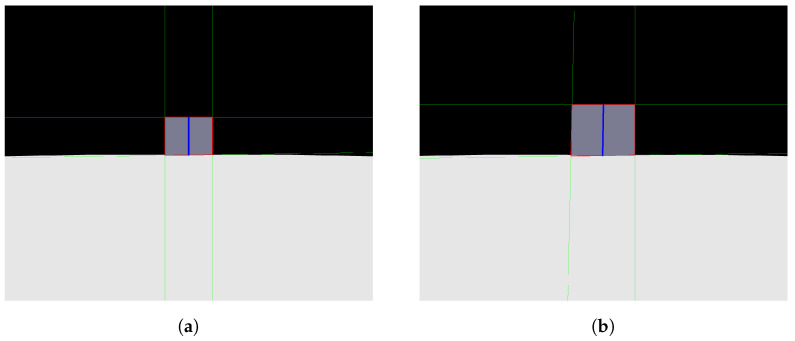
Analysis obtained through image processing algorithm. (**a**) Vision sensor first photo analysis; (**b**) vision sensor second photo analysis.

Having validated the image processing algorithm created, the next step is to apply it within the CoppeliaSim software, chosen for the complete simulation of the system. To proceed, it is necessary to set up a scene in which there is a camera that moves linearly and with a view parallel to the step, capturing two photos along the way. In the CoppeliaSim software, it is necessary to opt for a vision sensor, a component that is capable of capturing the necessary images and being integrated into the model assembled in the simulator. A code in Python language was written so that the vision sensor performed a movement as described previously while capturing the necessary photos. Figure 9a shows the first photo captured by the vision sensor within the software, while Figure 9b shows the second photo captured. The figures show the CoppeliaSim environment, where there is a platform on which objects are placed for interaction. The visual sensor is in front of the step, on top of the platform. The environment also has a black background so that there is a contrast with the platform and objects. In Figure 10a,b it is possible to see the result of the image processing algorithm when finding the edges of the front face of the step in the captured photos. Using the edges found in both photos, the measurements of interest were calculated and a step height of 1.0056 m and a step distance of 3.9020 m were obtained. The original measurements configured were 1.0 m for the step height and 3.9 m for the step distance, showing that the results were even more accurate when compared with the images from the simulated camera. The largest error obtained was 0.56 percent. In this way, the algorithm and method can be considered approved for application in conjunction with the robot.

### 3.2. First Climbing Algorithm

The first movement algorithm defined was using the pitch–pitch combination. Previously, the robot’s complete movement algorithm was developed in [18], where success was achieved with the proposed simulations, and the model was successfully validated. Therefore, in this work, we started from the state in which the previous movement algorithm was left.

The new study began by adding the visual sensor to the robot, which was attached to the front of the first module (“head” module). This fixation can be seen in Figure 1, with the sensor represented as a blue dot. With the sensor fixed, movement and photo-capture tests began and a standard was established. The complete logic for the defined movement sequence was explained in detail in Section 2.1, topic Section 2.2.1, as well as all the parameters used and the pre-established conditions for the case addressed.

**Figure 11:**
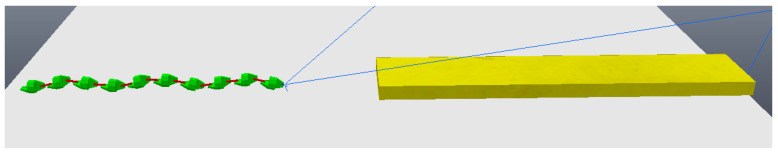
Start of movement with robot still far from the step.

**Figure 12:**
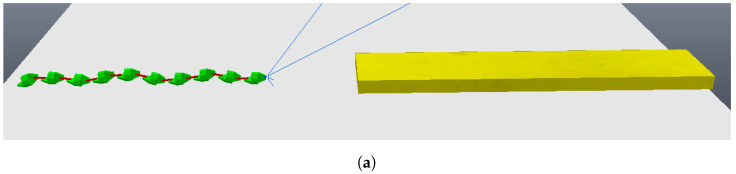

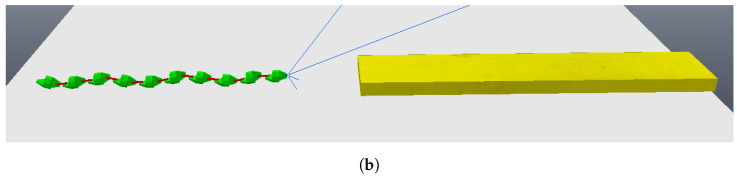
Moments when the photos were taken. (**a**) First module parallel to the step taking the first photo; (**b**) first module parallel to the step taking the second photo.

**Figure 13:**
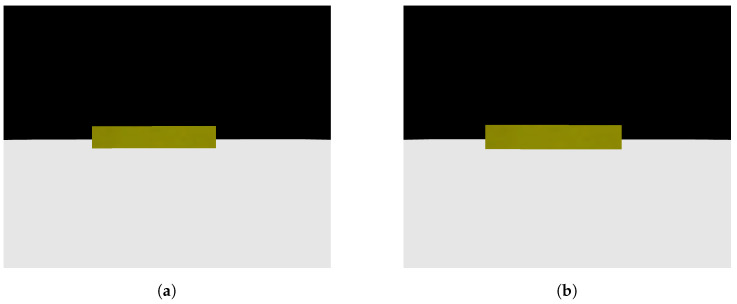
Photos taken by the robot during the algorithm. (**a**) First photo taken; (**b**) second photo taken.

**Figure 14:**
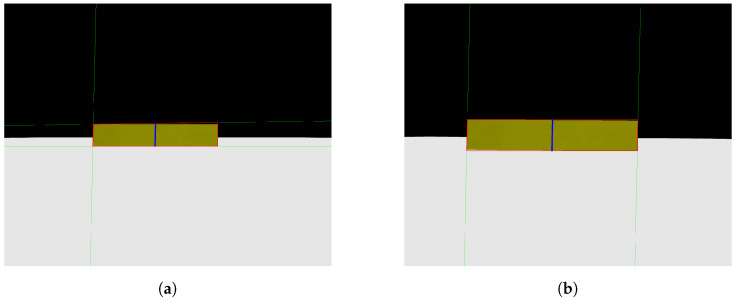
Photos analysis—image processing. (**a**) Edges found for the first photo; (**b**) edges found for the second photo.

**Figure 15:**
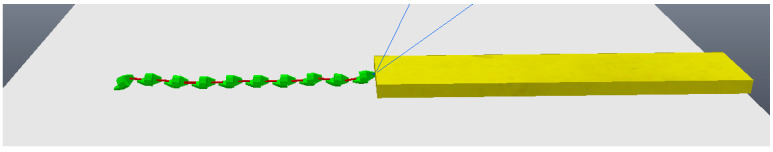
Robot reaches the step.

**Figure 16:**
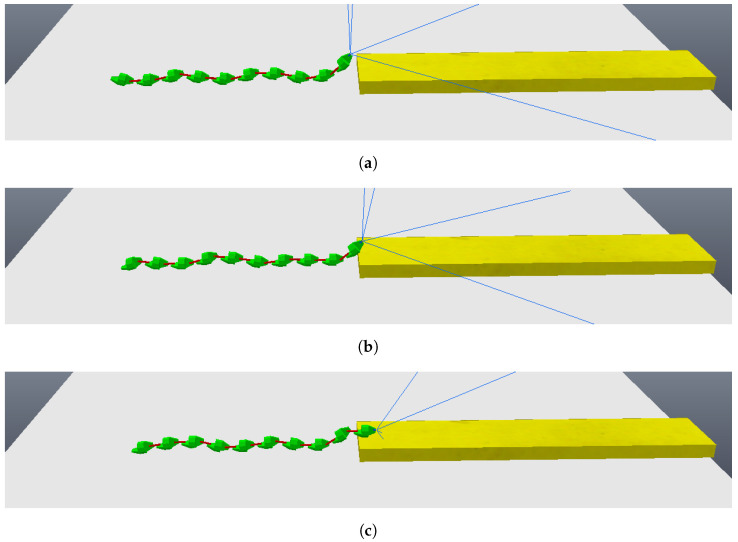
First three movements to climb the step. (**a**) Lifting the first module; (**b**) robot goes forward and the first module touches the corner of the step; (**c**) lifting the second module and supporting the first module on top of the step.

**Figure 17:**
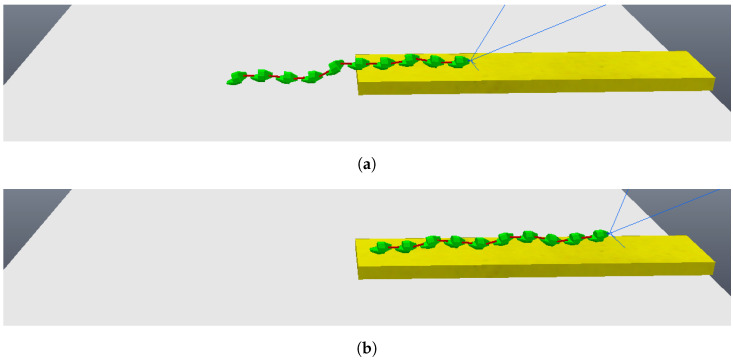
Middle and end of climbing the step. (**a**) Robot halfway to complete the climbing; (**b**) climbing completed.

The simulation begins with the robot moving towards the step, although still far away, as can be seen in Figure 11. The robot then stops its movement and aligns its first module (“head”) so that it is parallel to the front face of the step, subsequently capturing a photo, as shown in Figure 12a (photo captured shown in Figure 13a). Then, the first module returns to its position prior to alignment and moves again towards the step, following the same previous movement. After a pre-determined amount of cycles of the robot’s movement have passed, it stops again, aligns its first module with the front face of the step, and captures another photo, as shown in Figure 12b (photo captured shown on Figure 13b). The robot then returns its frontal module to the position before alignment and resumes its movement as it had done after the first photo was captured. From then on, with the values of the step height and the distance to the step calculated (from image processing—Figure 14), the robot continues moving until it is as close as possible to the step (Figure 15). Upon reaching the limit of horizontal movement towards the step, the robot starts to lift the first module (module closest to the step), as shown in Figure 16a. When lifting the module, a space appears between the robot and the step, and therefore the robot moves closer to the step, supporting the module that was lifted on the corner of the step (Figure 16b). With the first module supported on the step, the second module is lifted while the first is adjusted on top of the step (Figure 16c). Then, the robot follows the same movement sequence until it is completely on top of the step, approaching the step as much as possible, lifting the module closest to the step that is still below, and adjusting the module(s) that already are on top. Figure 17a shows the robot halfway to completing the climb, a position in which there is an exchange of the modules that lead the movement. When the robot is still mostly at the bottom, the modules at the bottom lead the approach to the step, passing the lead to the modules that are at the top of the step when the robot becomes mostly at the top of the step. Figure 17b shows the robot at the end of the climb, completely on top of the step.

### 3.3. Second Climbing Algorithm

The second movement algorithm defined was to use the pitch–yaw combination. In the same way as the previous algorithm, the complete algorithm for this form of movement was previously developed in [18], where success was achieved with the proposed simulations, and the model was successfully validated. Therefore, in this work, we started from the state in which the previous movement algorithm was left.

The changes began by adding the visual sensor to the robot, which was attached to the front of the first module (“head” module), just like was done before. Furthermore, the robot was positioned at some distance from the step so that it was possible to capture images of the front face of the step.

**Figure 18:**
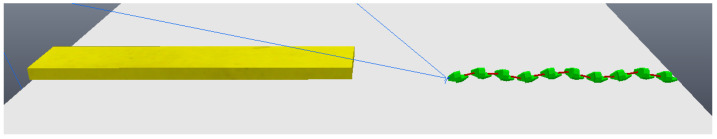
Start of movement with robot still far from the step.

**Figure 19:**
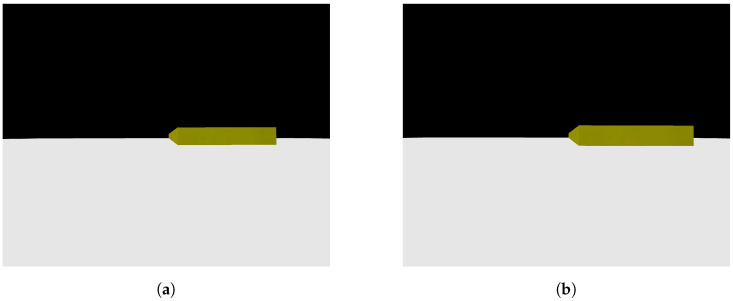
Photos taken by the robot during the algorithm. (**a**) First photo taken; (**b**) second photo taken.

**Figure 20:**
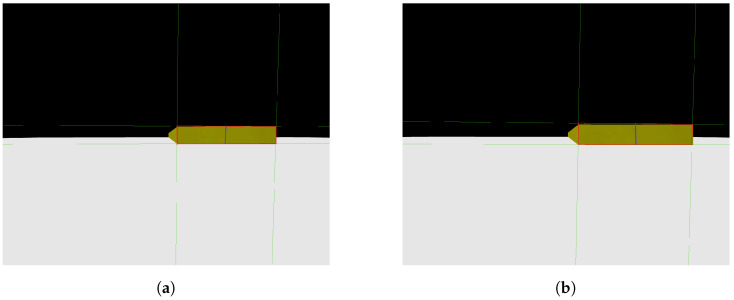
Photos analysis—image processing. (**a**) Edges found for the first photo; (**b**) edges found for the second photo.

**Figure 21:**
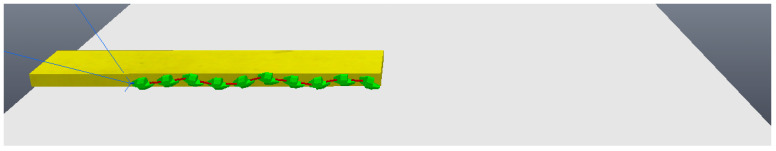
Robot reaches the side of the step.

**Figure 22:**
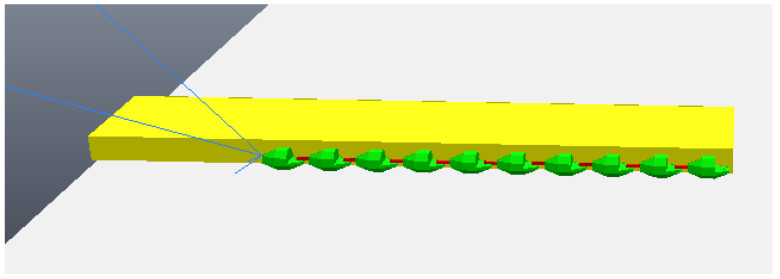
Robot aligns its joints to the step.

**Figure 23:**
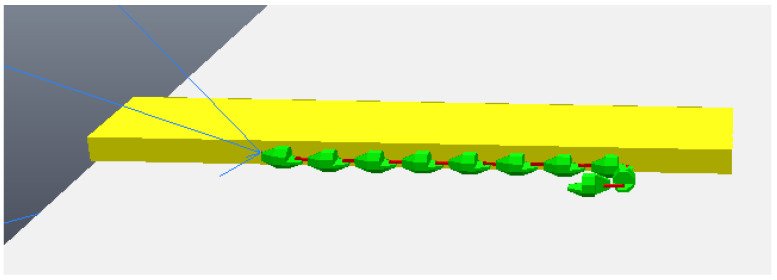
Robot make a base.

**Figure 24:**
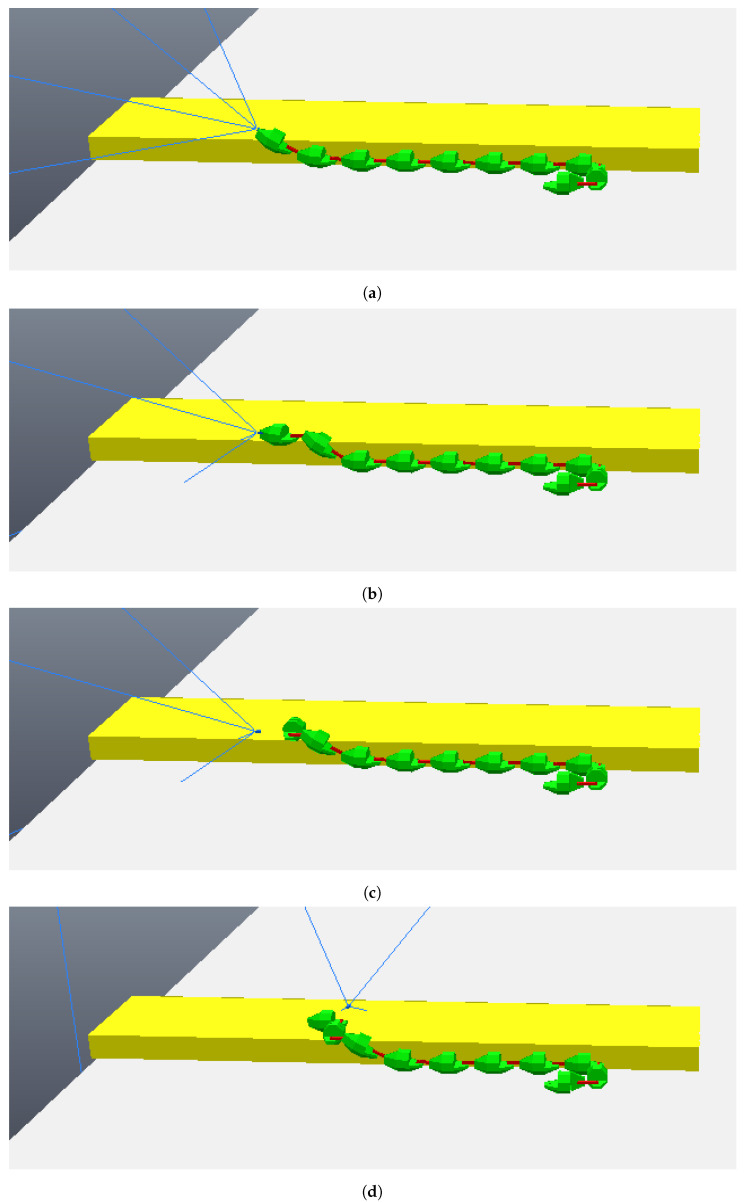
First four movements to climb the step. (**a**) Lifting the first module; (**b**) lifting the second module and aligning the first; (**c**) first module yaws to be on the top of the step; (**d**) third module lifts, second module descends aligning, and the first module yaws also aligning.

**Figure 25:**
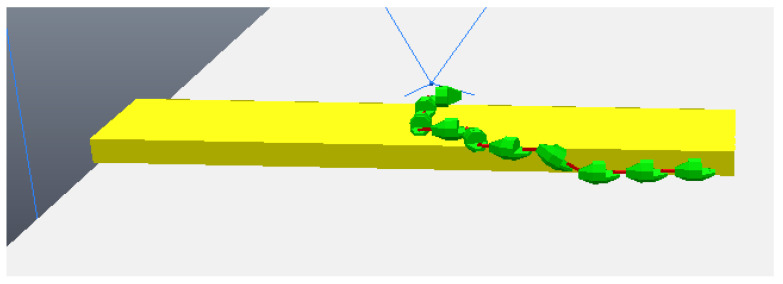
Robot makes the base at the top and undoes the base at the bottom.

**Figure 26:**
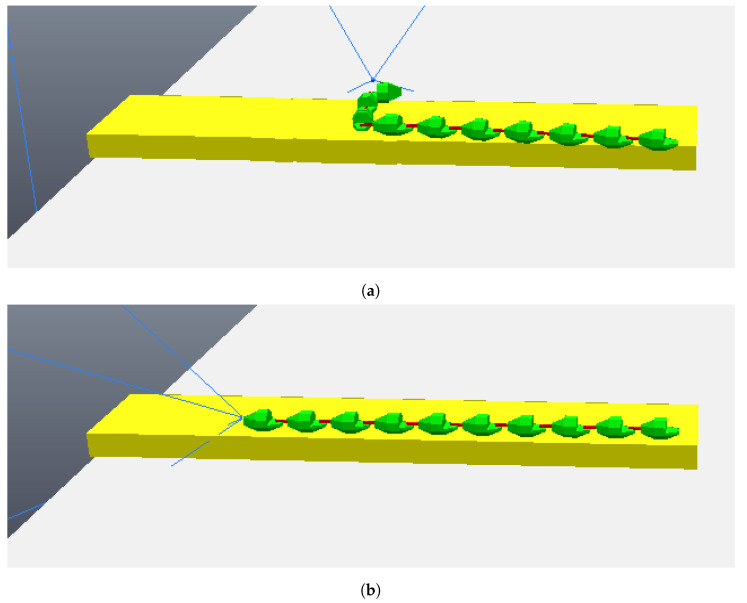
End of climbing the step and aligning all the modules. (**a**) All of the robot is on top of the step still with the base; (**b**) aligning all the modules on top of the step.

The simulation begins with the robot moving towards the step, although still far away, as can be seen in Figure 18. As before, the robot makes two stops while going towards to the step and captures two different photos, so the algorithm of image processing could calculate the height of the step and the distance the robot is from the step. These photos are shown in Figure 19. With the values of the step height and the distance to the step calculated (from image processing—Figure 20), the robot continues moving until it is next to the step, already leaning sideways, with its length parallel to the step, as shown in Figure 21. At this point, the robot aligns all its joints so the robot position becomes completely parallel to the step (Figure 22). Next, the robot needs to create its support base to be able to continue with the climbing algorithm. The two final modules (opposite the “head” module) rotate in a yaw motion until they form a U-shaped base (Figure 23). In the same way, as mentioned in the previous algorithm, the frontal module (“head”) is tilted upwards (pitch movement), up to a better degree based on the step height (Figure 24a), followed by two consecutive movements, where the second module pitches upward until reaching the same degree that the first module reached, while the front module pitches downward until it returns to a vertical position (Figure 24b). Then, the front module yaws until it is supported on the step at 90 degrees to the previous position (Figure 24c). The third module then begins to rise while the second module descends and the first module yaws to align itself on top of the step (Figure 24d). This sequence is followed until it is possible to create a base on top of the step similar to the one created below, as shown in Figure 25. After creating the base, the robot’s support is now on top of the step and the sequence described previously is continued until the climb is completed (Figure 26a). Finally, the robot undoes its base and returns to a stretched position, parallel to the length of the step, as can be seen in Figure 26b.

### 3.4. Rotation

**Figure 27:**
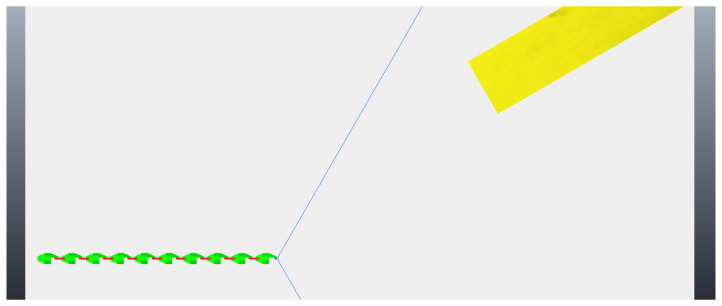
Aligned robot joints before movement starts.

**Figure 28:**
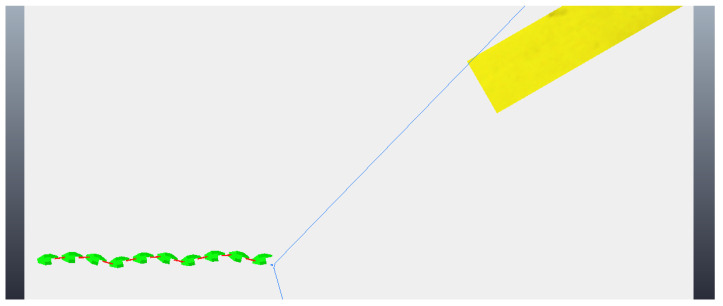
Start of rotation movement.

**Figure 29:**
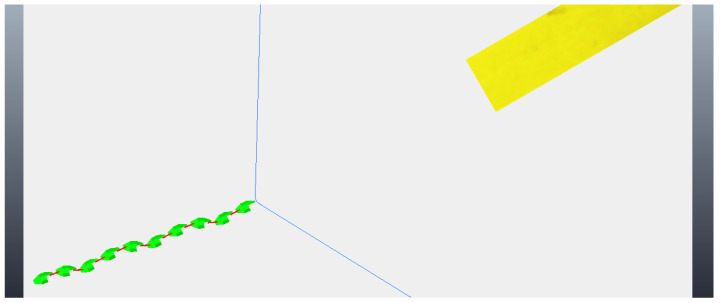
Middle of the rotation process.

**Figure 30:**
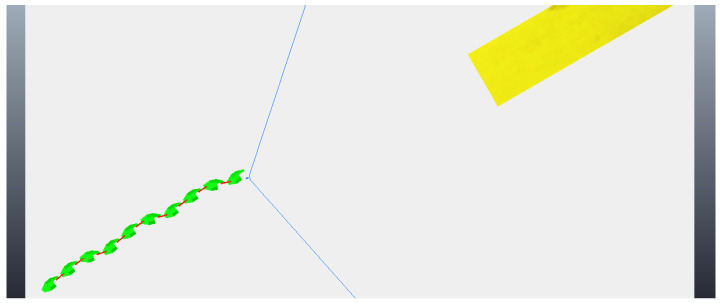
Rotation movement finished, and robot aligned to the step.

**Figure 31:**
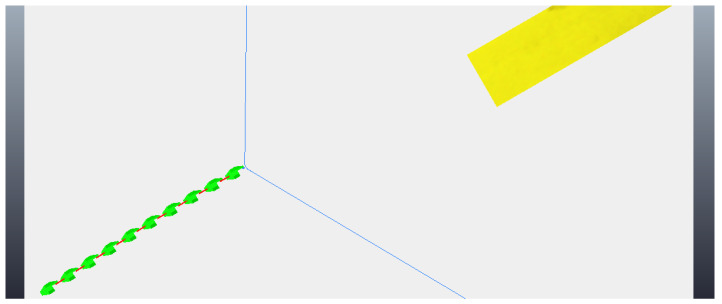
Robot joints aligned before starting a climbing algorithm.

As mentioned previously, the snake robot needs to be aligned with the step for the climbing algorithms to work correctly, preventing the robot from falling off the obstacle during climbing. Therefore, for the robot to have freedom of movement, it is necessary to rotate its position around the z-axis, an axis orthogonal to the plane of the floor. Figure 27 shows the robot with its joints aligned to begin rotational movement. The rotational movement is initialized, as shown in Figure 28, followed by the rotational movement reaching its middle, as shown in Figure 29. Figure 30 shows the instant at which the rotation is completed, at which point it is necessary to align the joints again to correctly proceed to a climbing algorithm. Figure 31 shows the joints aligned and the robot aligned with the step in front, ready to initiate a climbing movement.

## 4. Discussion

In this work, two algorithms for climbing prismatic step-like obstacles were developed and demonstrated in the Coppelia robotics simulator, along with a perception technique for identifying the height of the obstacle and its distance to the camera based on line detection for shape identification and matching.

Aiming to demonstrate that snake robots can climb vertical objects similar to steps, the techniques presented were developed catering to the proposed problem in the two very specific scenarios presented, where the initial position and orientation of the robot to the obstacle are set in two distinct configurations and the obstacle has a very specific shape. Although the robot is capable of rotating and aligning itself to the initial position necessary to follow the climbing algorithms, the specificity of the proposed obstacle is a limitation to be addressed in future work, aimed at generalizing the problem of navigation in three-dimensional environments, although in man-made urban environments obstacles such as the step used in the simulations are frequently encountered [45].

The vision algorithm utilized employed a few priory assumptions about the shape and orientation of the obstacle that does not apply to a more general scenario. Also, visual pollution and the problem of segmentation were neglected, considering a simplified simulation environment or experimental setup where the obstacle was visible in a homogeneous background, without confounding factors (other objects visible in the scene or textures in the background) that could negatively affect the results. Still, the technique demonstrated employed simplified locomotion planning in order to acquire the necessary images, traversing perpendicular to the faces to be measured, something that could be perfected with a more sophisticated algorithm such as structure from motion to reduce uncertainty about the surrounding environment in future works. Similar approaches are described in the literature in which the null space policies are adjusted to render more information from the camera motion, as described in [37], where active structure from motion was employed.

When comparing the simulations of the two climbing algorithms, some differences are noticeable. The first algorithm simulation took 10 min and 31 s. The first part of the algorithm, until the robot reached the step, took 2 min and 7 s, that is, the part in which the robot climbed the step took 8 min and 24 s. Moving up the step in pitch–pitch takes extra time due to the need to become closer to the step with each module that goes up; however, only when going up do the modules perform extensive movements, and at all other times the movement is short. The second algorithm simulation took 14 min and 40 s. The first part of the algorithm, until the robot reached the side of the step, took 9 min and 9 s, that is, the part in which the robot climbed the step took 5 min and 31 s. This algorithm does not have extra time due to the need to become closer to the step with each module that goes up; however, there are many extensive movements to align all the modules during the algorithm, spending extra time.

## 5. Conclusions

In this paper, two climbing algorithms were proposed and validated using the Coppelia robotics simulator, on top of a vision-based algorithm that identifies and parameterizes an obstacle in the shape of a step.

For image processing, a Python code was developed to simulate camera images capturing the front face of the step. The algorithm successfully identified the edges of the step’s front face, allowing accurate calculation of its height and distance from the robot. This algorithm was validated both in a simulated environment and within the CoppeliaSim software, demonstrating high accuracy in calculating step parameters.

Regarding the first climbing algorithm utilizing the pitch–pitch combination, simulations showed that the robot successfully climbed the step with an acceptable error rate. The algorithm efficiently aligned the robot’s modules and adjusted their movements to navigate the step. Despite taking over 10 min to complete the simulation, the algorithm demonstrated effective step-by-step climbing.

Similarly, the second climbing algorithm, which employs a pitch–yaw combination, demonstrated the robot’s ability to climb steps using an alternative method. Although the simulation time was longer than that of the first algorithm, it exhibited robustness in climbing steps with precise module alignment.

The possibility of the snake robot to make rotations on the plan is important since it allows the robot to analyze the environment autonomously through computational vision and define which is the best method of climbing, aligning with the obstacle in the best way possible to follow the chosen algorithm. Thus, the environment in which the robot may be inserted becomes broader and more diverse.

Therefore, it is clear that the contribution of this work lies in the combination of locomotion techniques (pitch–pitch and pitch–yaw) with computer vision to climb specific obstacles. Applying a classic computer vision technique, such as the Hough transform, specifically to improve a modular robot’s ability to detect and climb obstacles is an adaptation that adds practical value. While each of these techniques may exist separately, integrating them in a coordinated way to solve a specific climbing problem has innovative elements. This work serves as an important starting point that can be improved upon with future research, offering a foundation upon which new techniques and improvements can be developed to further increase the effectiveness and adaptability of modular robots in more complex environments.

In conclusion, the experiments conducted validated both the image processing algorithm and the movement algorithms within the CoppeliaSim environment. The results obtained are promising and demonstrate that snake robots are suitable for tasks involving navigation in environments where planar movement is inadequate, such as environments where there are obstacles like steps. More work needs to be done to obtain more general path planning and mapping algorithms for complex scenarios.

## Figures and Tables

**Table 1:** Table of relative errors.

Case Number	Measurement	True Value	Estimated	Relative Error
Case 1	Distance	60	59.709	0.004
Case 1	Height	300	299.011	0.003
Case 2	Distance	65	64.467	0.008
Case 2	Height	300	297.137	0.009
Case 3	Distance	60	59.531	0.007
Case 3	Height	350	345.398	0.013
Case 4	Distance	65	64.201	0.012
Case 4	Height	350	343.597	0.018
Case 5	Distance	70	68.809	0.017
Case 5	Height	400	392.347	0.019
Case 6	Distance	80	79.704	0.003
Case 6	Height	500	499.179	0.001

## Data Availability

Data are not available for privacy reasons.

## Abbreviations

The following abbreviations are used in this manuscript:

ACM	Active Cord Mechanism
POAL	Perception-driven obstacle-aided locomotion
SFM	Structure From motion
SLAM	Simultaneous localization and mapping
